# The N1–N2–LPC Pattern in Processing Advertising Pictorial Metaphors: An ERP Study

**DOI:** 10.3389/fpsyg.2018.02566

**Published:** 2018-12-18

**Authors:** Shuo Cao, Yanzhang Wang, Hongjun Chen, Huili Wang

**Affiliations:** ^1^Faculty of Management and Economics, Dalian University of Technology, Dalian, China; ^2^School of Foreign Languages, Dalian University of Technology, Dalian, China

**Keywords:** pictorial metaphor, ERP, N1, N2, LPC

## Abstract

We investigated what the temporal processing is of advertising pictorial metaphors. After presenting “a word of product” and “its advertising pictures,” the experiment instructed participants to make a follow-up true–false judgment considering what the picture intended to suggest. A repeated-measures ANOVAs for a 2 (picture type: metaphor, non-metaphor) × 2 (prime–target condition: congruent, incongruent) × 3 (electrode site: Fz, Cz, Pz) experimental condition was conducted on three components, N1 (100–150 ms), N2 (200–300 ms), and LPC (400–600 ms and 600–1,000 ms). The results show that metaphor pictures elicited larger amplitude in N1 (broadly distributed), N2 (frontally biased) and LPC (parietally biased), roughly reflecting an entire process with an initial response to visual onsets, an early recognition of semantic violations and a prolonged reanalysis process of semantic integration. We argue that, different than verbal metaphors, this faster processing occurred due to the involvement of visual pathway.

## Introduction

Pictorial metaphor is defined as depicting an object in terms of a different kind of object to which it bears a resemblance (e.g., mountains depicted as rooftops) (Dent-Read et al., 1994). The cognitive processing of metaphors, as Lakoff et al. (1988) suggest, involve cross-domain mapping between the target and the source. It was reported to involve the activation of the whole brain (Chen and Cheng, 2013). For this complicated processing, some theoretical models have been proposed, including the Hierarchical Hypothesis (Grice, 1975; Swinney and Osterhout, 1990; Tang et al., 2017), the parallel hypothesis (Harris, 1976; Blasko and Connine, 1993; Yang et al., 2013), and the context-dependent hypothesis (Pynte et al., 1996; Glucksberg, 2001, 2003). Recently, the Career of Metaphor Model (Bowdle and Gentner, 2005) identified the advantages of comparison operation for the novel metaphors versus a more automatic categorization process for conventional metaphors. Basically, it is believed that the processing of metaphors go through the stages of recognition, semantic integration and self- generated thought (Clark and Lucy, 1975; Clifton, 1983; Whittock, 1990; Cieślicka, 2006). Moreover, the processing pictorial metaphor is assumed to be related with typology of pictorial metaphors (van Mulken et al., 2010). Hybrid metaphors (versus contextual metaphors and similes) in adverting pictures (Forceville, 2007), as the fusion of two objects, demands the most cognitive effort with regard to the perceived complexity and deviation from expectation (van Mulken et al., 2014).

Despite the fact that metaphor terms are represented or cued by graphic means in pictorial stimuli, the study of pictorial metaphor is more complex and has been focused on the identification of neural mechanism mainly by differentiating it from those of verbal metaphors and/or of literal images (Carroll, 1994), a research line that follows the evidence that the pattern of neural activation in picture recognition tasks is different from that of matched words (Moore and Price, 1999; Chee et al., 2000; Bright et al., 2004; Gates and Yoon, 2005; Reinholz and Pollmann, 2005). According to the Dual Coding Theory (Paivio and Csapo, 1971; Paivio, 1986, 2010), this occurs because verbal and pictorial stimuli trigger two functionally independent, but interconnected, multimodal systems, with one specialized for non-verbal stimuli, which directly represents the perceptual properties and affordances of non-verbal objects and events.

To study the neurocognitive mechanism of pictorial metaphors, different methods have been attempted, such as eye tracking (Indurkhya and Ojha, 2013), EEG (Ma et al., 2016), fMRI (Ojha et al., 2017) and distributional method of semantics (Bolognesi, 2016). Comparatively, ERP is advantageous in terms of high time-resolution and its application to explore the temporal course and dynamic mechanism of pictorial metaphor (Kutas and Hillyard, 1980; Hillyard and Kutas, 1983), which is of great significance to the study of advertising visual stimuli, as the typical observation time for print advertising is two seconds in an editorial context (Pieters et al., 1996). The analysis of what occurs in the split of time with brain’s response to advertising visual stimuli can contribute to the development of both the cognitive study and marketing research of pictorial metaphors.

However, the ERP findings obtained from the extant experiments of pictorial metaphors in the context of advertising are not consistent, even if N400 is often found to reflect both a conceptual integration demanding more cognitive effort and a gradient of difficulty in semantic integration (van Berkum et al., 1999; Coulson and van Petten, 2002; Coulson, 2008; Holt et al., 2009; van Berkum, 2009; Davenport and Coulson, 2011; Kutas and Federmeier, 2011). Ortiz et al. (2017) was successful in identifying P200 in an early time-window and a late P600 and the current density of the cerebral activity based on an observation of surface sLORETA source of the cerebral activity. It was pointed out that more brain activity in the right brain was detected for the processing of hybrid pictorial metaphors contrasted with non- metaphor visual stimuli. However, based on the smaller volume of experiment trials (only 12 items) resulting in lower signal noise ratio for the ERP wave forms, the working hypothesis and conclusion need further investigations. The electrophysiological exploration of pictorial metaphor has also aroused interest in China. Ma et al. (2016), in a task of match judgment for vehicle pictures and animal words, provided the evidence that inappropriate pictorial metaphors elicited larger N300 amplitude than appropriate ones. He attributed the enhanced N300 to the activation of image-specific system.

The controversy over the findings calls for a further exploration regarding the temporal course of the processing of pictorial metaphors. What needs to be taken into consideration here is that pictorial metaphors have picture-superiority effect (Defeyter et al., 2009), specifically in the task of recognition memory. This advantage over words, according to Paivio (1991), is a result of semantic information encoded by two separate routes, namely, pictures are processed via both an image pathway and a verbal pathway. We propose that this two-pathway processing is likely to facilitate pictorial metaphors to be processed temporally still earlier. Based on the previous literature considering metaphor processing and visual processing, the study aims to examine three potential components, which are presumed to be highly relevant to the processing of pictorial metaphors, N1, visual N2 and LPC.

Many previous studies showed that visual N1, observed around 150–200 ms post-stimulus, is an early visual response to visual stimulation. The N1 is elicited by visual stimuli, and is part of the visual evoked potential, a series of voltage deflections observed in response to visual onset, offsets, and changes. The earliest N1 component can be detected frontally detected around 100–150 ms (Luck, 2005). Its amplitude is influenced by selective attention and has been used to study a variety of attentional processes (Miniussi et al., 1999; Griffin et al., 2002; Lange et al., 2003; Doallo et al., 2004; Correa et al., 2005; Doherty et al., 2005). In the current experiment, participants should recruit cognitive efforts by paying more attention to the metaphor pictures before being able to recognize the target and the source, and the later linking of part or whole of the two items. So it is hypothesized that in the initial stage of attention, N1 should be detected in amplitude change.

Meanwhile, another cognitive marker of processing pictorial metaphor is proposed to be N2. N2 follows a prominent temporo-occipital negative peak at around 180 ms in the visual modality. The extant findings show that it is modulated by the detection of novel stimuli and to the orienting of visual attention, including a fronto-central (anterior) component related to the detection of novelty or violation from a perceptual template when the eliciting stimuli are attended. N2 was more sensitive to the degree of perceptual deviation (Demiralp et al., 2001; Polich and Comerchero, 2003). To effectively drive the novel N2, visual stimuli must be either highly unfamiliar and thus deviate from long-term context or deviate considerably from short-term context. In addition, when novel stimuli had no predictive value, they elicited an N2 largest over frontal and central scalp. In the current study, located in the center of the visual field, the visual stimuli are definite to be attended. All these polarly unfamiliar visual stimuli were screened based on a pretest of familiarity, indicating that in some degree the priming word is not predictive to the upcoming picture for the item. Accordingly, it is hypothesized that metaphorical pictures should elicit an enhanced N2 for metaphor pictures, possibly in the frontal area.

In addition, LPC (P600), modulated in amplitude by the novelty of the metaphoric meaning, as revealed in a consistent manner by the extant studies of metaphors, is another important index worth examining in the study. It’s a positive- going event-related brain potential (ERP) component with a peak between 600 and 800 ms after stimulus onset, generally found to be largest over parietal scalp sites (relative to reference electrodes placed on the mastoid processes). Linked with recollection and retrieval, it is thought to play a crucial role in reflecting a continued analysis (or reanalysis), either at the linguistic level of input that produced the violation (Kuperberg et al., 2007), or a complete reanalysis of the input (Kolk and Chwilla, 2007; Van de Meerendonk et al., 2009, 2010). The effect of LPC in some studies was significantly higher for novel metaphors than for the rest of the expression types which is counter-intuitive since novel metaphors require more integration in order to be comprehended (Coulson and van Petten, 2002; De Grauwe et al., 2010; Weiland et al., 2014). However, other results do not support this conclusion (Arzouan et al., 2007a; Coulson and van Petten, 2007; Goldstein et al., 2012). These studies, due to differences in task type (including semantic judgment tasks, reading tasks, or delayed response procedures) and stimuli selection criteria with levels of meaningfulness, metaphoricity, familiarity and cloze probability controlled to various degrees), show a result with reduced LPC amplitudes to novel metaphors versus literal and anomalous utterances. Brouwer et al. (2012) and Rataj et al. (2018) believed this difference was attributed firstly to the greater efforts related to the integration of semantic information retrieved from the dissociation and identification of the source and the target observed in time window such as N400; and secondly to the recollection of the entire stimuli as the experiment tasks involve, which also recruit more effort in the case of novel metaphors. The prolonged retrieval of information from implicit memory and mapping two distantly associated items (termed by Friederici et al., 1999) as secondary semantic integration processes may overlap with what initially occurs in the time window of N400, resulting in a reduced LPC amplitude to novel metaphors. One of the objectives of the study is to study what is the LPC amplitude for pictorial metaphors, enhanced or reduced. We believe that the more effort invested in collecting and retrieving information from memory for the analogical mapping will lead to an increase in LPC amplitude. What is unique with the current study is that instead of focusing on N400, it suggests that the discrepancy detected of the source and the domain should occur earlier and be reflected in N2 time window due to the involvement of visual processing. So it is less possible to observe an overlapping between the initial and the late semantic integration.

Based on the above-mentioned findings, this study employs ERP technology to examine the temporal course of the processing of advertising pictorial metaphors by comparing visual advertising pictures with literal images. Since the extant ERP studies showed that N1, N2, and LPC index some aspects of semantic processing of visual stimuli, we would expect these three neural components to occur in the current study with enhanced N1, N2, and LPC for metaphorical pictures. We propose a faster and more complicated processing mechanism represented by an enhanced N1–N2–LPC pattern for pictorial metaphors relative to literal images.

## Materials and Methods

### Participants

A total of 19 right-handed volunteers (11 males, 8 females; mean age = 31 ± 7.46 years) participated in the ERP experiment. They all had normal or corrected-to- normal vision and had no history of psychiatric or neurological disorders as established by self-report. This study was carried out in accordance with the Declaration of Helsinki, and written informed consent was secured from all participants. The protocol was approved by the Biological and Medical Ethics Committee of Dalian University of Technology, China.

### Stimuli

**FIGURE 1:**
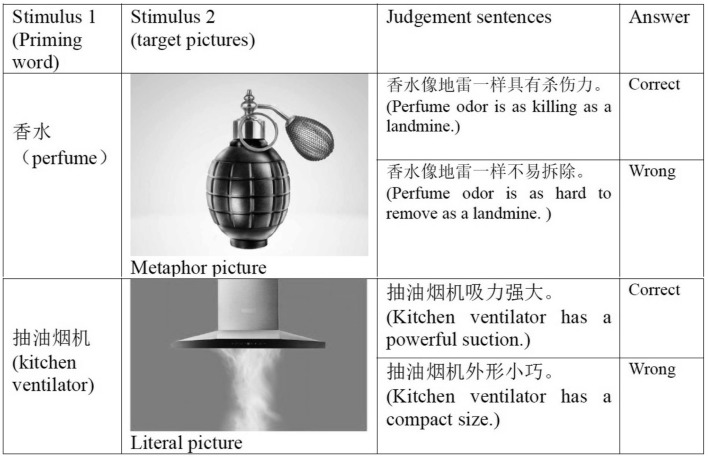
Examples of the stimuli used in this study.

The experiment stimuli include 120 prime product names in Chinese, 60 target advertising pictures and 120 corresponding Chinese sentences conveying what the corresponding pictures intended to suggest (Figure 1 shows representative examples). There were two types for pictures: metaphor pictures of hybrid- structures and literal pictures of products; two prime–target conditions: congruent (when product names match target pictures) and incongruent (when products names don’t match target pictures); two outcomes for sentence judgment: appropriate and inappropriate.

The picture stimuli were selected as follows: out of 132 advertisement pictures from China’s markets of various media, 62 were selected. Based on a preliminary test with a five-point Likert scale ranging from 1 (the least familiar) to 5 (the most familiar), 31 less familiar pictures were retained, as familiarity has a modulating influence on the metaphor processing (Blasko and Connine, 1993; Giora and Fein, 1999; Giora, 2003; Blasko and Kazmerski, 2006; Schmidt and Seger, 2009). It was carried out among 84 undergraduates in Dalian University of Technology, China, who did not participate in the later ERP experiment. Then, another 31 non-metaphor advertisement pictures, across similar product types and with similar number of pictures for each type, were used to counterbalance the experimental stimuli. Finally, the stimulus set included 30 metaphor pictures and 30 non-metaphor pictures, plus 2 pictures (one metaphor picture and one non-metaphor picture) used for practice. All the pictures were in the same luminance, shade and size, specifically with colors removed by Adobe Photoshop 13.0 in consideration of the metaphorical implications colors have.

A pretest of matching between prime words and target pictures was carried out before the experiment. A group of five students participated in a naming test for the items involved in the representation of pictorial metaphors of advertising. The average agreement rate was 93%. For the controversial namings, a second round of discussion was conducted with more participants or the corresponding pictures were discarded.

The product names, spanning the top sellable product categories (cars, electronic appliances, personal care, food and beverage). The lexical frequency of these names was assessed in *The CCL Corpus of Chinese Texts* to ensure that participants could comprehend the stimuli in the most commonly used terms. For instance, contrasted with 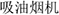 (of 16 lexical entries), 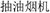, which was found labeled with up to 130 entries, was adopted in naming kitchen ventilators due to its high lexical frequency.

In the judgment statements of pictorial metaphor, the syntactic structure is “A is as X as B.” X in the appropriate statement referred to the perceived shared feature intended by the advertisement, while in the inappropriate statements, it was one of the unmapped features belonging to the source item, so that a polarized contrast was made with regard to metaphor judgment. Meanwhile, the sentence length remained generally similar, falling in the range of 7–14 characters.

The products (the target) and the related referent (the source) in the statements were named basically in consistent lexical length of Chinese characters to avoid the redundant processing of either each in the mapping continuum. For example, in the statement illustrating a pictorial metaphor in which shoes are presented like a comfortable bed, 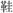 (shoes) is paired with 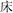 (bed), both in one-Chinese character, as two-Chinese- character 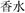 (perfume) with 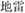 (landmine) in a perfume-landmine metaphor picture. All the Chinese characters were in Song typeface with the same font size.

### Procedure

**FIGURE 2:**
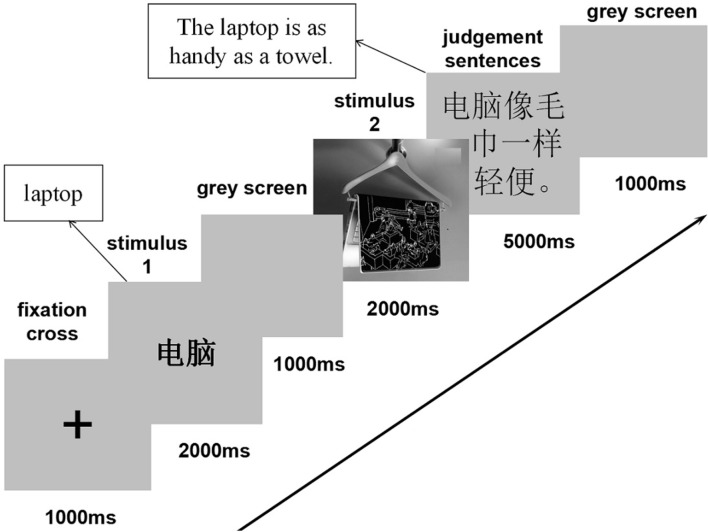
Experimental procedure (The English in squares is the translation for the Chinese stimuli).

We used the stimulus1 (prime) – stimulus 2 (target) experimental paradigm as previously used in non-verbal semantic processing studies (Federmeier and Kutas, 2002; Hamm et al., 2002). After the stimuli presentation, participants were asked to answer follow-up true–false comprehension questions. Figure 2 summarizes the experimental procedure. The first stimulus was the word of a product, and the second was its advertising picture. The word-picture pairs were presented randomly and once. There were two blocks with 60 trials each. All the stimuli were presented in the center of screen. In each trial, after an initial “+,” which was presented for 1,000 ms, appeared a word for the advertised product with a presenting duration of 2,000 ms. After that, a 1,000 ms black screen was presented before a statement conveying what the picture intended to suggest. The participants were asked to respond as quickly as possible in the duration of 5,000 ms by pressing 1 for the correct and 2 for the incorrect. There was another black screen occurring for 1,000 ms before the next trial. The participants conducted a brief exercise with four trials before the experiment.

### Data Acquisition

Electroencephalogram data was continuously recorded (band pass 0–100 Hz, sampling rate 1,000 Hz) with The Cognionics Quick-20 DC amplifier (Cognionics, Inc., San Diego, CA, United States). As a preliminary study, we only recorded three electrode sites based on 10-20 electrode montage although we used an electrode cap. The ground electrode was placed on the forehead and left mastoid was used as recording reference. Two mastoid electrodes were additionally applied for subsequent offline referencing.

ERP data were analyzed using the software of EMSE. Ocular artifacts were corrected with ICA. Spectral baseline correction was conducted and segments contaminated with artifacts exceeding amplitude of ±100 μV were rejected from averaging. The averaged ERPs were low-pass filtered with a 30 Hz low pass filter (24 dB/OCT).

On the basis of the research purpose and related similar experiments, N1, N2, and LPC, as well as corresponding electrode sites, Fz, Cz, and Pz were measured and analyzed. N1 was observed for peak and latency by analyzing its data between 100 ms and 150 ms. N2 and LPC were examined for the mean amplitude due to the unclearness/ambiguity of the peak in most participants. N2 was defined in the time-window of 200–300 ms after stimulus onset and LPC in two time windows, 400–600 ms and 600–1,000 ms. A repeated-measures ANOVAs for a 2 (type: metaphor, non-metaphor) × 2 (condition: congruent, incongruent) × 3 (electrode site: Fz, Cz, Pz) experimental conditions was conducted on three components. Greenhouse–Geisser corrected *p*-values when the degrees of freedom are higher than 1.

## Results

The analysis of the behavioral results shows a higher accuracy rates (>96%) and therefore, it is reliable that the subjects apprehended the metaphorical meanings.

**FIGURE 3:**
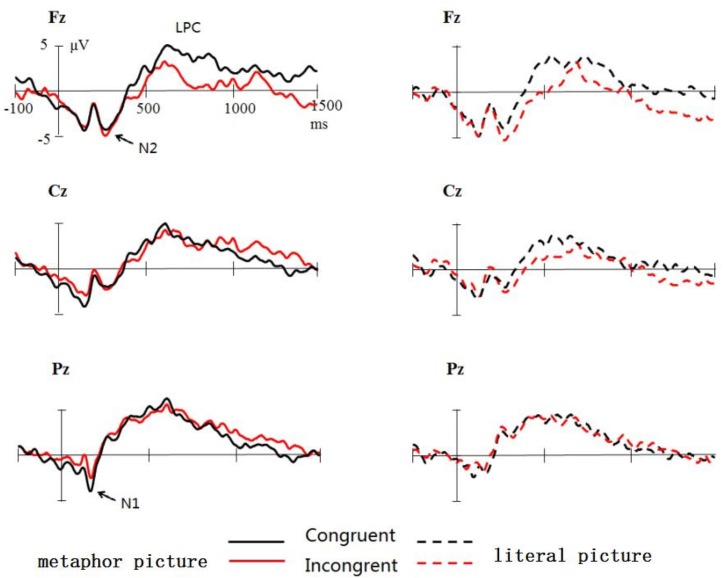
Grand average ERP waveforms elicited by pictures in two conditions: congruent (black) and incongruent (red) from Fz, Cz, and Pz.

**FIGURE 4:**
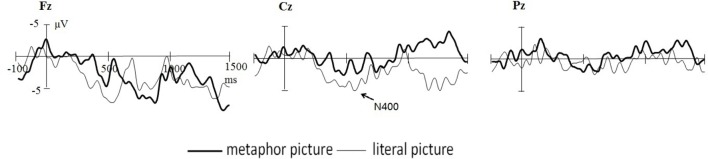
Grand average ERP waveforms elicited by metaphor pictures (black) and literal images (red) from Fz, Cz, and Pz.

Figures 3, 4 show the total average ERPs original waveform and differences generated by two different conditions (incongruent minus the congruent conditions). It can be clearly seen that metaphor pictures and literal pictures induce N1, N2, and LPC. Relative to the incongruent condition, the congruent condition elicited significantly enhanced LPC, especially for the area of central frontal in the case of literal pictures.

### The N1 Results

The mean latency of N1 is 122 ms, demonstrating an insignificant main effect across type, condition, and multiple interaction effect (*p* > 0.1). ANOVA results of N1, however, did reveal the significant main effect of electrode side [*F*(2,36) = 19.569, *p* = 0.000, $\eta_{p}^{2}$ = 0.512], with the largest amplitude occurring at Fz (-4.1 μV), followed by Cz (-3.1 μV) and Pz (-2.1 μV). Interestingly, the interaction of metaphor type and congruent condition was significant [*F*(1,18) = 6.163, *p* < 0.05, $\eta_{p}^{2}$ = 0.188], indicative of that N1 (-3.9 μV) generated in metaphor type for the congruent condition was significantly larger than that of incongruent condition [-2.6 μV, *F*(1,18) = 4.864, *p* < 0.05, $\eta_{p}^{2}$ = 0.213] whereas no such difference was observed in the literal pictures. Other effects were not found significant (*p* > 0.1).

### The N2 Results

ANOVA results of N2 reflected that the interaction effect did reach significant [*F*(1,18) = 5.863, *p* < 0.05, $\eta_{p}^{2}$ = 0.188] despite an insignificant main effect of metaphor type and condition (*p* > 0.05). Follow-up analysis by contrasting two conditions demonstrated that in metaphor type N2 has no significant effect on the congruent connotation (congruent: -1.6 μV; incongruent: -1.6 μV; *p* > 0.1), whereas in for literal pictures, incongruent conditions (-1.9 μV) elicited significantly larger N2 (*p* < 0.05) than congruent conditions (-1.3 μV). Meanwhile, analyses of data yielded a significant main effect of electrode sites [*F*(2,36) = 45.054, *p* = 0.000, $\eta_{p}^{2}$ = 0.715], N2 at Fz being the largest in amplitude (-5.1 μV) and Pz (2.8 μV) the smallest. It was found that the interaction of electrode side and metaphor [*F*(2,36) = 11.447, *p* = 0.000, $\eta_{p}^{2}$ = 0.389] and that of electrode site and congruent [*F*(2,36) = 8.411, *p* = 0.002, $\eta_{p}^{2}$ = 0.318] were both significant. The significant effect was also found with the three-dimension interaction between metaphor type and congruent condition and electrode site [*F*(2,36) = 5.988, *p* = 0.006, $\eta_{p}^{2}$ = 0.250]. What is noticeable is that further analysis showed a highly significant interaction effect of metaphor and congruent at Fz (*p* < 0.03).

### The LPC Results

#### 400–600 ms

In the time window of 400–600 ms, the main effect of metaphor type was found significant [*F*(1,18) = 4.608, *p* < 0.05, $\eta_{p}^{2}$ = 0.456], a larger LPC amplitude (4.2 μV) in metaphor type than in literal type (3.4 μV). The main effect of electrode sites was also significant [*F*(2,36) = 7.339, *p* = 0.002, $\eta_{p}^{2}$ = 0.290], the amplitude at Pz being the largest (5.3 μV) and that at Fz the smallest (2.9 μV). Despite that the main effect did not approach significance [*F*(1,18) = 1.248, *p* = 0.287, $\eta_{p}^{2}$ = 0.366], it is revealed that there was a significant effect between metaphor type and congruent condition [*F*(1,18) = 6.356, *p* < 0.05, $\eta_{p}^{2}$ = 0.347]; importantly, the three-dimension interaction was also significant [*F*(2,36) = 4.605, *p* < 0.05, $\eta_{p}^{2}$ = 0.582]. The analysis of interaction effect performed for every electrode site reflected that at Fz and Cz there was significant interaction effect between metaphor type and congruent condition (*p* < 0.05), indicating that the LPC in metaphor type did not differ for the congruent condition (Fz: 3.2 μV; Cz: 3.8 μV) from for the incongruent condition (*p* > 0.1), but in literal type, it is significantly enhanced for congruent condition (Fz: 4.4 μV; Cz: 3.6 μV) contrasted with incongruent condition (Fz: 1.0 μV; Cz: 1.7 μV) (*p* < 0.05).

#### 600–1,000 ms

Analyses of this time window indicated the main effect of electrode sites was approaching significance [*F*(2,36) = 3.134, *p* = 0.056, $\eta_{p}^{2}$ = 0.148], with the largest LPC amplitude in parietal Pz (3.6 μV), significantly larger than those in frontal Fz (2.6 μV, *p* < 0.05) and central Cz (2.6 μV, *p* < 0.05). Despite of the insignificant main effect of both metaphor and congruence (*F*s < 1), the interaction effect was found very significant both between electrode sites [*F*(2,36) = 4.894, *p* < 0.05, $\eta_{p}^{2}$ = 0.224] and metaphor and between electrode and congruence [*F*(2,36) = 5.212, *p* < 0.05, $\eta_{p}^{2}$ = 0.141]. It is further found that in the central and parietal area (Cz and Pz) metaphor (3.6 μV) elicited larger amplitude of LPC than literal metaphors (2.6 μV) (*p* < 0.05). In the frontal area (Fz), congruent conditions induced larger amplitude of LPC (3.9 μV) than incongruent conditions (1.2 μV, *p* < 0.03). No other significant effect was found (*p* > 0.1).

## Discussion

**FIGURE 5:**
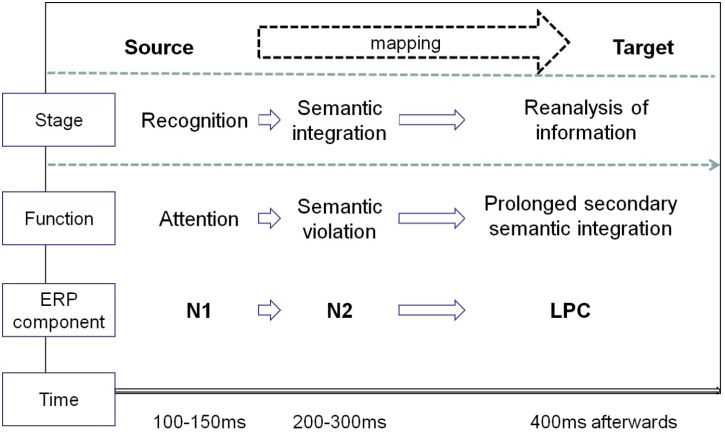
The temporal course in processing pictorial metaphors.

In the current study, advertising pictures of pictorial metaphor and literal images were used to investigate the neurocognitive process involved in visual metaphor comprehension. The main purpose was to identify the possible temporal processing of pictorial metaphors in the context of advertising, by observing the time window of hypothetically relevant ERP components and comparing the results with those previously reported in related metaphor studies. As predicted, the time window of three potential components effectively demonstrate the overall temporal processing of pictorial metaphors, which initiates with a perceptual response, followed by a violation-based semantic integration based on a visual mismatch violation and a late reanalysis of information retrieved from memory. Due to the involvement of visual pathway in the metaphorical processing of visual modality, the recognition stage and semantic integration occur earlier and the reanalysis prolonged. The current study, at least in part, has opened the possibility of outlining the entire processing mechanism of visual pictorial metaphors (see Figure 5).

For the entireness of the temporal course of pictorial metaphors processing, N1 was analyzed and found with an amplitude change. As both metaphor and literal pictures elicited N1, this response to visual onsets detected in such an early time window is likely to be attributed to various perceptual stimulation of the experiment pictures, such as shape (Luck et al., 1994; Luck and Yard, 1995).

In both metaphorical pictures and literal pictures, more negative N2 was found in incongruent condition, which is consistent with the previous findings (Suwazono et al., 2000). They found that when the priming metaphor picture did not reveal predictive value for the following judgment task, the amplified violation detection elicited a N2 largest over frontal and central scalp (novel N2). Its occurrence is based on high unfamiliarity and relatively complex shapes of the stimuli. As aforementioned, instead of using simple-shape black and white drawing, we used the decolored real-life advertising pictures. The visual N2 effect occurred in the study due to the unfamiliarity of the stimuli themselves and that resulting from the experimental paradigm. The pictorial metaphors used in the current study are chosen after a pretest of familiarity, which may ensure the retained unfamiliar pictures are very deviant from long-term memory representations. On the other hand, the effect of novelty in incongruent condition with the priming word and an unexpected picture is also possible to evoke the N2, since it is sensitive to the degree of perceptual deviation. For the example aforementioned, when the prime word for laptop is followed by an unusual pictorial metaphor represented by a cross-category combination of a towel (a toiletry) and a laptop (an electronic product), a violation occurs of what participants stereo-typically perceive of the two items and it is sensitively detected by N2 effect.

Some may argue that this N2 effect is similar to MMN. As far as the current study is concerned, visual N2 and visual MMN are not related. N2 is identified to be activated frontally when observed for the analysis of attention and semantic processing, while in general visual MMN is elicited temporo-occipitally. We think that visual N2 and visual MMN involve two different processing mechanisms. Instead, we argue that this frontally biased N2 novel effect for pictorial metaphors may be very much like the effect of N400 for verbal metaphor (Salmon and Pratt, 2002), marking an early semantic integration. This finding is helpful in providing neural evidence for the argument that verbal metaphors and pictorial metaphors might share the same cognitive mechanism, but different in that the involvement of visual pathway facilitates a faster conceptual integration of pictorial metaphors, reflecting a larger difficulty of conceptual retrieval and integration and leading to more effort related to the integration of the unrelated items for the conceptual expansion. N2 was less reported in most previous metaphors including either verbal stimuli or pictorial stimuli. The occurrence of N2 in the current study suggests that the comprehension of pictorial metaphors involves an additional image-based semantic processing, as verified in the study by Balconi and Amenta (2010) that mental imagery plays a role in the cross-domain mapping in metaphor comprehension.

Besides the N2 effect, the findings show that metaphor conditions evoked a larger amplitude of LPC than non-metaphor conditions, similar to earlier studies (Coulson and van Petten, 2002; De Grauwe et al., 2010; Weiland et al., 2014). But different from previous studies reporting increased late positivity, such as those which investigated familiar metaphors (De Grauwe et al., 2010), neither familiar nor unfamiliar metaphors in the normative studies preceding the EEG experiments (Weiland et al., 2014), or not explicitly pretested metaphors on familiarity or conventionality scales (Coulson and van Petten, 2002), the study examined the least familiar visual pictures. All these studies, to some extent, support the argument that conventionality plays a modulating role in the complicated processing of pictorial metaphors.

Indexing recovery and integration of additional material from semantic memory with enhanced costs involved in the mapping process, LPC is observable for metaphor pictures (400–600 ms and 600–1,000 ms), mainly due to the recruitment of greater effort for metaphor pictures related to the integration of semantic information retrieved within the N2 time window with the preceding context. Especially when the priming word is consistent with the metaphor picture, a successful cross-domain mapping is conducted by making a feature-by-feature comparison, which will tax more attention on participants’ side (Daffner et al., 2000). In this late processing stage, for the example of laptop-towel (shown in Figure 2), the violation that occurred early from the visual linking of the laptops and the towel is solved based on more sustained cognitive effort. Participants may now conceive that the linking does make sense by realizing that the two items share in common some features, both perceptually and conceptually, such as being compatible, thin, light, and even nice-looking. Besides, the follow-up true–false comprehension questions in the study demands a recollection of the whole picture before making a judgment, which is very likely to prolong the integration of semantic information. In addition, the results showed the literal-metaphor difference was largest over the midline parietal site (Pz), which was in line with the findings reported by Voss and Federmeier (2011) and Rataj et al. (2018).

However, the previous report of an enhanced N400 (Arzouan et al., 2007b) for metaphors is not focused in the study. Because we analyzed the amplitude of the early stage LPC, 400–600 ms post stimulus onset, that is, incongruent condition elicited more negative going component. Actually this time window is the N400 component. It can be explained that the discrepancy/violation perceived between the target and the source occurred and was observed in N2 time window. This may be attributed to the involvement of visual pathway for the processing of pictorial metaphors. According to Forceville (2002, 2007), in the visual modality of metaphorical representation, some features need not to be retrieved from memory as they have been given visually. This advantage of multi-sensory perception may facilitate the analogical mapping process. The recruitment of image-based system is what makes pictorial metaphor different than verbal metaphors. On the other hand, LPC was also found in late time window (600–1,000 ms), suggesting an extension of violation solving as N400 does in the processing of verbal metaphors. In some sense, LPC identified in the current study, with an earlier perceived violation and a later reanalysis of semantic integration, may have functioned for pictorial metaphors very much alike N400 for verbal metaphors.

In a nutshell, this N1–N2–LPC pattern might roughly indicate a specific process of pictorial metaphors, since it is not observed in the studies of verbal stimuli processing. However, it only occurs to the hybrid-structure of pictorial metaphors and it is not less generalized to all types of pictorial metaphors. This needs to be further explored, especially to explore whether there exists a gradient of N2 effect in the processing of visual metaphors of different types as that of N400 for verbal metaphors.

## Conclusion

The current study pointed to the possible neurocognitive processing of pictorial metaphors of advertising pictures by comparing it with that of literal pictures. The results identified a specific pattern for visual metaphors, characterized by an initial perceptual response (N1), an early semantic violation (N2), and an enhanced reanalysis process (LPC). This faster processing of visual metaphors is important to the field of advertising marketing. When target products are visually advertised in metaphors, they are likely to be processed in a facilitating manner. Especially in the late stage of processing, the much effort related to and invested in the collection and retrieval of information enables the consumers to have an in-depth comprehension of the item advertised before making smart purchase decisions. It will be more interesting to study the pictorial metaphors in other contexts for its complete analysis of cognitive mechanism.

## Author Contributions

All the authors designed the research. SC collected the data. SC and HW analyzed the data and all authors interpreted the data and wrote the manuscript.

## Conflict of Interest Statement

The authors declare that the research was conducted in the absence of any commercial or financial relationships that could be construed as a potential conflict of interest.
